# Integrated evaluation of environmental monitoring, rheumatoid arthritis biomarkers, and genetic variants in textile dyeing workers

**DOI:** 10.1186/s42506-026-00217-2

**Published:** 2026-06-29

**Authors:** Amal Saad-Hussein, Eman M. Shahy, Mona M. Taha, Gehan Moubarz, Heba Mahdy-Abdallah, Lamia S. Ellaithy, Inas A. Saleh, Atef M.F. Mohammed

**Affiliations:** 1 Environmental & Occupational Medicine Department, Environment & Climate Change Research Institute, National Research Centre, Cairo, Egypt; 2 Air Pollution Department, Environment & Climate Change Research Institute, National Research Centre, Cairo, Egypt

**Keywords:** Textile dyeing workers, Rheumatoid arthritis biomarkers, Air particulates, Air volatile organic compounds, *CYP2E1* gene polymorphism, *GST* gene polymorphism

## Abstract

**Background:**

Workers in the textile dyeing industry are occupationally exposed to high concentrations of particulates and volatile organic compounds (VOCs). This work aimed to study the environment-gene interaction as a risk factor for rheumatoid arthritis in textile dyeing workers.

**Methods:**

This cross-sectional comparative study included 140 exposed male workers and 130 matched control workers. Air monitoring of chemical air pollutants in the workplace was conducted for 12 months. Estimation of anti-CCP, CRP, RF, ANA antibodies, and the *CYP2E1* and *GST* genes polymorphisms was studied in both groups.

**Results:**

High air concentrations of PM_10_, SO_2,_ H_2_S, and VOCs were detected in the preparatory and dyeing sections compared to the printing section. Anti-CCP, CRP, RF, and ANA were statistically higher in exposed workers compared to the control group. Anti-CCP and RF in preparatory workers were significantly higher compared to printing workers. Multivariate analysis revealed interaction in *CYP2E1*,* GST* polymorphism and different environmental exposures on RF.

**Conclusion:**

The environment-gene interaction of the elevation of PM10 and VOCs concentrations in the dyeing section, with the *CYP2E1* (*C2/C2* mutant genotype) and the *GST* (*M1* polymorphism), could be a risk factor for RF elevation in the workers from the dyeing section compared to the other two sections. The key recommendations include improving environmental control in the workplace, ensuring the proper use of personal protective equipment (PPE), conducting pre-employment screening for genetic susceptibility through genetic counseling for CYP2E1 and GST polymorphisms, and providing regular medical follow-up for workers carrying the CYP2E1 *(C2/C2)* genotype and the GST *(M1)* allele.

**Supplementary Information:**

The online version contains supplementary material available at 10.1186/s42506-026-00217-2.

## Introduction

Rheumatoid Arthritis (RA) is a common chronic inflammatory autoimmune disease that causes progressive joint damage and, without treatment, can result in serious disability [1]. RA affects synovial joints (small and large) in approximately 1% of adults worldwide [2]. Although the exact causes of RA are still unknown, it is believed that it arises from a combination of genetic and environmental factors [3]. It was proven that both genetic and environmental factors contribute to the development of RA, with genetic differences accounting for about 50% of the variability in risk [4].

Several studies have focused on the relationship between air pollution and RA [5, 6]. Sigaux et al. [6] suggested that air pollution could be a risk factor for RA, through the induction of an inflammatory response and oxidative stress. Occupational exposure to cotton dust has been associated with an increased risk of developing RA [7, 8], as the textile industry is one of the oldest industries that includes a variety of processes.

Textile dyeing represents the final processes applied to textiles before sent to the consumers. Sellappa et al. [9] mentioned that workers in the dyeing processes of textiles are frequently exposed to multiple chemicals, which vary with time and process. The first step in textile dyeing is the bleaching operation, which releases chlorine dioxide, and fabric printing releases hydrocarbons and ammonia, and fabric-finishing operations can also release formaldehyde into the air [10, 11]. Therefore, textile dyeing processes usually include the use of boilers, which generate particles with different sizes, and gases such as SO_2_, CO, NO_2_, chlorine vapor during the bleaching process, while petroleum, formalin, and acid acetate vapors are usually generated from the pigments [10, 11]. Gupta et al. [11] mentioned that most generators used in dyeing processes contribute to the release of PM directly into the air, and it also emits SO_2_ and NO_2_, which transform into secondary particulates in the atmosphere. It was also found that volatile organic compounds (VOCs) are also produced when textile materials containing lubricating oils, plasticizers, or other materials, that can volatilize or thermally degrade into volatile substances, when subjected to heat [10, 11]. Thus, long-term occupational exposure or accidental overexposure can potentiate occupational health hazards in the exposed workers in the textile dyeing industry.

Genetic susceptibility to specific environmental chemicals increases the risk of developing health hazards in susceptible individuals [12]. Cytochrome *P450 2E1* (*CYP2E1*) is involved in the activation of chemicals, such as benzene, while *Glutathione S-transferase mu1* (*GSTM1*) and *glutathione S-transferase theta 1* (*GSTT1*) are involved in its detoxification, and are mapped on chromosome 1 (*1p13.3*) and chromosome 22 (*22q11.2*), respectively. There is evidence that some allelic variants are associated with differences in the detoxification efficiency [13].

Identification of genetic susceptibility could have an important role in risk assessment studies. It could replace the traditional methodologies for determining hazardous chemicals. The link between these genes and chemical pollutants is required to define the role of genetic polymorphism in the toxicity and hazardous impacts of these environmental pollutants [14].

The aim of this work was (1) to describe the chemical environmental pollutants in the textile dyeing industry, including printing, dyeing, and preparatory workplaces, (2) to study the environment-gene interaction (*CYP2E1*,* GSTM1*, and *GSTT1* gene) as a risk factor for the elevation of the RA biomarkers.

## Methods

### Study design and participants

A cross-sectional exposed and non-exposed comparative study was conducted in 2022-2024 on 140 male workers (Exposed group) in three textile dyeing sections (printing, dyeing, and preparatory departments) from a textile dyeing factory in Cairo, Egypt. They were occupationally exposed to textile dyeing processes for more than 5 years, and their age between 25 and 50 years. All workers were on morning shift for 8 h/day, 6 days/ week. None of the included workers was using personal protective equipment. They are not exposed to cotton dust, as their workplaces are away from the textile industrial processes. They also didn`t previously work in dusty jobs; this was the first job for all the included workers.

The control workers were 150 non-exposed males chosen with the same sociodemographic criteria and residing away from the industrial zones (Control group). But after excluding the control workers above 50 years, 130 workers were included.

The two groups were matched in their age range, social residential areas, educational levels, and their salaries were within the same range. There was no family history of RA, all the workers were within the normal weight, and were doing moderate physical work.

### The industrial operation process passes through 3 steps: Dyeing, processing, and printing

#### The dyeing process

- Closed dyeing: the dyeing process runs inside a closed system, so emissions of vapor dyes are less than those of the open system.
- Open dyeing: the dyeing process occurs in an open system. Most of the emissions (VOCs and other gases) are from the open process, in the form of vapor dyes beside the heat.
- Both open and closed processes are in the same ward.

#### Preparatory or processing

- It is done to give the cloth its final texture. It occurs in 3 places, and each place has 2 lines of processing. The length of the line 30–40 m, it enters the beginning of the cloth piece to be withdrawn and processed. Processing is done by water vapor and heat, so emissions (particulate matter with different sizes and gases) at this site are due to both heat and vapor.

#### Printing

- The first step is sampling (screen printing): where testing occurs for the printed cloth with the shapes and patterns required. After printing, the frame of the screen is washed with warm water and kerosene. The area of the sampling room is small and has one exhaust ventilation instrument. So, it is nearly a closed process. Emissions in this step are VOCs and other gases.
- The second step: It is the main printing ward (roller printing). Each machine contains a cast iron cylinder fitted with a nylon sheet with the patterns to be printed. It is connected by tubes to the barrel dyes, fixatives, and solvents (kerosene) in the presence of variable temperatures according to the type of cloth. After printing, fixation of colors, drying inside the machine, and change of barrel dyes are done. Washing of the tubes of the machine and the cylinders with water and kerosene is done. Emissions in this step are VOCs, other gases, and particulate matter of different sizes.

### The Exposures

The floors are wetted with water, kerosene, liquid dyes, and fixatives. There are stored barrels of dyes and kerosene. Moreover, the ventilation of the place is bad, and there are no exhausts.

- Dyes: color dyes.
- Water and water vapor.
- Kerosene and other solvents and their fumes.
- Other chemicals: acids, bases, inhibitors.
- Heat.
- The printing section has the largest number of workers as the process needs continuous observation. Therefore, workers in the printing process have the heaviest exposures.

### Environmental measurements

Environmental monitoring of the air in the workplaces of the included factory was bimonthly for 12 months. Particulate matter with different fraction sizes (TSP, PM_10_, and PM_2.5_) was estimated in the air of the three textile dyeing sections in an Egyptian textile factory (5 locations in the printing section, 6 locations in the preparatory section, and 4 locations in the dyeing section) using CEL-712 Casella Micro-Dust Pro Particulate Monitor. Also, monitoring of the gaseous pollutants emitted from textile dyeing processes in the same locations, such as VOCs, NO_2_, SO_2_, NH_3_, CO, and H_2_S, was done using Aeroqual’s 200 portable air quality monitors.

### Questionnaire

Personal interviews were done with all the included subjects to fulfill personal, past history, and family history of autoimmune diseases, and medical history, including symptoms and signs of acute and chronic autoimmune diseases, and hospitalization for fever or chronic diseases. The questionnaire also thoroughly asked about exposure to environmental and occupational pollutants. The 2010 American College of Rheumatology (ACR) / the European Alliance of Associations for Rheumatology (EULAR) (ACR/ EULAR) diagnostic criteria for clinical or subclinical RA diagnosis were also included in the questionnaire [15].

### Clinical examination

Medical examination of large and small joints was done for the included workers to detect symptoms and signs of RA.

### Laboratory investigations

A 5 ml venous blood sample was collected from each participant. A portion of the blood was placed in a clean, dry tube and centrifuged to separate serum. The separated serum was kept in the fridge until analyzed. The rest of the blood was collected in EDTA tube for genetic study.

The following parameters were performed in serum for the diagnosis of RA:

- Rapid Latex test for the qualitative screening and semi-quantitative determination of Rheumatoid Factor (RF) [16] and C- Reactive Protein (CRP) [17]. RF was considered to be positive (+ve) if it was above 10 IU/ ml, and for CRP if it was above 10 mg/ L. Manufactured for MG Science& Technology Center “STC” www.biostc.net.
- Determination of Anticycliccitrullinated peptides antibodies (Anti- CCP), using the ELISA technique according to DiaMetra S.r.l. Headquarters: Via Garibaldi, 18 – 20,090 SEGRATE (MI) Italy. Email: info@diametra.com. According to the pamphlet, “Since no international reference preparation for anti-CP antibodies is available, the assay system is calibrated in relative arbitrary units.” According to the pamphlet, the normal range in serum samples from healthy blood donors, the following ranges have been established with the Anti CP test: AntiCPIgG (AU/mL): Negative < 10, Positive > 10.
- Determination of Antinuclear antibodies (ANA) using ELISA technique according to ORGENTEC Diagnostika GmbH, Carl-Zeiss-Straße 49 -51, 5529 Mainz, Germany. According to the pamphlet, ANA (U/mL): Negative < 1.0, Borderline between 1.0 and 1.2, Positive > 1.2.

### Genetic studies

DNA extraction was performed from peripheral blood through QIAamp^®^ DNA Blood kit (Qiagen, Hilden, Germany) following the manufacturer’s protocol for genetic analysis of *GST* gene and *CYPE1* gene polymorphisms.

Screening of Glutathione-S-transferase (*GST*) polymorphisms (detoxification gene) was done using polymerase chain reaction (PCR) for prediction of susceptibility according to Adibhesami et al. [18].

GSTM1: Primers used were as follows: (P1), *5′-CGC CAT CTT GTG CTA CAT TGC CCG-3′; (P2)*,* 5′-ATC TTC TCC TCT TCT GTC TC-3′*; and (P3), *5′-TTC TGG ATT GTA GCA GAT CA-3′*. P1 and P3 amplified GSTM1, while P1 and P2 served as an internal control for each PCR reaction.

GSTT1: primers used were *5′-TTC CTT ACT GGT CCT CAC ATC TC-3′*, and *5′-TCA CCG GAT CAT GGC CAG CA-3′*.

Determination of genetic polymorphisms of *CYP2E1*.

CYP2E1 genotyping was determined according to Moghadas et al. [19], by polymerase chain reaction–restriction fragment length polymorphism (PCR-RFLP).

For CYP2E1 genotype, Forward primer (*5′-CCC GTG AGC CAG TCG AGT-3′*), and the Reverse primer (*5′-ATA CAG ACC CTC TTC CAC-3′*).

### Statistical analysis

The collected data were statistically analyzed using SPSS version 23. Quantitative data were expressed as means and standard deviations (SD), and the categorical data were represented as numbers and percentages. For comparisons between the exposed and control groups, an independent t-test and Pearson’s Chi-square (χ2) were used for continuous and categorical data, respectively. The likelihood ratio was used for categorical comparisons when more than 25% of the expected cells were less than 5. Analysis of variance (ANOVA) and Least Significant Differences (LSD) as a Post-hoc test, as well as Pearson’s Chi-square (χ2), were used in the comparisons between workers in the three different working places (preparatory, dyeing, and printing areas). Because the sample size of the subgroup of workers according to the gene polymorphisms was small, the Kruskal–Wallis test was used to compare biomarker levels. A multivariate general linear model (GLM) was used; the dependent variables were normally distributed. The coding scheme for the GLM included fixed risk factors such as the type of occupational exposure (controls and workers from the three sections), smoking status, *CYP2E1* polymorphism, and *GST* polymorphism. Duration of exposure was treated as a random risk factor to account for variability in exposure-related risk. The difference was considered significant at a P-value < 0.05.

## Results

Table 1 shows no significant differences between the exposed workers and the controls in their ages and smoking index (SI). There was a significant increase in the joint pain complaints among the exposed workers compared to controls. Most of the joint pain complaints were pains of large joints, and only 3.6% of the workers were complaining of small joint pain, with a significant difference compared to controls. Clinical examination of the joints showed that exposed workers with abnormalities in joint examination were significantly higher compared to the control group. The highest abnormality was knee crepitus, followed by tenderness of large joints on pressure, and only 2 workers from the exposed group had elbow crepitus. 

Table (*Supplement 1*) shows significantly high concentrations of PM_10_ in the preparatory section compared to the printing and dyeing sections, and in the dyeing section compared to the printing section. Concentrations of the VOCs were significantly higher in the dyeing section compared to both the preparatory and printing sections, and in the preparatory section compared to the printing. Also, the air levels of SO_2_ were significantly higher in the preparatory and dyeing sections compared to the printing section,. Wwhile air levels of H_2_S in the printing section were significantly higher compared to the preparatory and dyeing sections.

**Table 1:** Comparisons of the personal data, joint complaints, and clinical examination between the exposed workers and the controls, Cairo, Egypt, 2022-2024

	Control (130)	Exposed (140)	*P*-value^a^
	Mean ±SD	Mean ±SD	
Personal data			
Age (years)	40.9±9.7	37±10.7	> 0.05
SI (pack-year)	3.3±0.6	3.2±1.1	> 0.05
	No. (%)	No. (%)	*P*-value^b^
Complaints			
Joint pain	15 (11.5)	58 (41.4)	< 0.0001**
Joint size with pain			< 0.0001**
- Large joints	14 (10.8)	53 (37.9)	
- Small joints (fingers/toes)	1 (0.8)	5 (3.6)	
Joint examination			
- Elbow crepitus	0 (0)	2 (1.4)	< 0.0001**
- Knee crepitus	9 (6.9)	48 (34.3)	
- Tenderness of large joint on pressure	1 (0.8)	9 (6.4)	

SI: smoking index= [(Cig per day *years of smoking) / 20], ^a^Independent t-test, ^b^likelihood ratio **: Significant at p<0.001

Table 2 shows a significant elevation of the anti-CCP in the exposed workers compared to their controls. The percent of positive CRP, RF, and ANA were significantly higher in the exposed workers compared to the controls.

**Table 2:** Comparisons of the autoimmune biomarkers between the exposed workers and their controls

Biomarkers	Control (130)		Exposed (140)		*P*-value^a^
	Mean ± SD		Mean ± SD		
Anti CCP (AU/mL)	8.08 ± 0.69		10.19 ± 0.29		0.004*
	No.	%	No.	%	*P*-value^b^
CRP (mg/L) (+ve)	0	0	17	12.1	< 0.0001**
RF (IU/ml) (+ve)	0	0	4	2.9	0.02*
ANA (U/mL)	1	0.8	13	9.3	0.002*
(+ve)					

^a^Independent t-test, ^b^likelihood ratio *: Significant at p<0.05 **: Significant at p<0.001

Table 3 shows that there are no significant differences in the age, duration of exposure, and SI between the workers in the three working sections (printing, dyeing, and the preparatory sections). Even ANOVA shows no significant differences between the three groups. LSD shows that Anti-CCP was significantly higher in workers from the preparatory section compared to the workers from the printing section. The frequency of positive RF workers was significantly higher among workers from the dyeing section compared to the other occupational groups. However, there was no significant difference in CRP and ANA between the workers in the three sections.

**Table 3:** Comparisons of the personal data and autoimmune biomarkers between the exposed workers according to their occupational tasks

Parameters	Workers from printing Sect. (35)		Workers from dyeing Sect. (87)		Workers from preparatory Sect. (18)		*P*-value^a^
	Mean ± SD		Mean ± SD		Mean ± SD		
Personal data							
Age (years)	37.8 ± 9.86		40.5 ± 9.96		43.1 ± 9.87		> 0.05
Exposure duration (years)	13.6 ± 1.17		15.0 ± 0.88		17.1 ± 2.03		> 0.05
SI (pack, year)	1.94 ± 0.71		2.85 ± 0.58		1.04 ± 0.53		> 0.05
Autoimmune biomarkers							
Anti-CCP (AU/mL)	9.12 ± 0.55^(a)^		10.41 ± 0.39		11.19 ± 0.80		> 0.05
	No.	%	No.	%	No.	%	*P*-value^b^
CRP (mg/L) (+ve)	6	17.1	10	11.5	1	5.6	> 0.05
RF (IU/ml) (+ve)	1	2.9	15	17.2	1	5.6	0.03*
ANA (U/mL)	2	5.7	8	9.2	3	16.7	> 0.05
(+ve)							

>10 is positive for Anti-CCP, CRP, and RF. >1.2 is positive for ANA

SI: smoking index= [(Cig per day *years of smoking) / 20],

N.B.: using LSD (a): significantly different from preparatory workers

^a^ANOVA, ^b^likelihood ratio *: Significant at p<0.05

Figure (*supplement 2a*) shows no significant difference in the distribution of *CYP2E1* polymorphism between the exposed workers and their controls (*Chi-square= 0.389*,*P*> 0.05). Figure (*supplement 2*b) shows non-significant differences in the distribution of *CYP2E1* polymorphism between the exposed workers from the three sections (*Chi-square= 2.233*, *P*> 0.05). Figure (*supplement 2*c) shows high significant difference in the distribution of *GST* polymorphism between the exposed workers and the controls (*Chi-square= 45.21*, *P*< 0.0001), as the percent of exposed workers with *GSTM1T1* polymorphism was higher than in the controls. Figure (*supplement 2*d) shows a non-significant difference in the distribution of *GST* polymorphism between the exposed workers from the three sections (*Likelihood ratio= 11.14*, *P*> 0.05).

Table 4 shows the multivariate analysis results of the different risk factors. There is significant interaction between *GST* polymorphisms and the mutation in the *CYP2E1* gene. RF was found to be significantly associated with the interaction of the polymorphisms of *the GST* and *CYP2E1* genes. Also, RF was significantly associated with the interaction of the two gene polymorphisms with the occupational tasks (representing the different of environmental exposures in the three sections).

**Table 4:** Multivariate analysis of the risk factors on the RA biomarkers

Source	Anti-CCP (AU/L)		CRP (mg/L)		RF (IU/ml)	
	F	Sig.	F	Sig.	F	Sig.
Corrected Model	0.69	0.93	0.86	0.74	1.60	0.02
Intercept	49.13	0.00	1.93	0.17	2.40	0.12
Duration of exposure (years)	0.17	0.68	0.09	0.77	0.28	0.60
*GST* polymorphism	0.19	0.91	0.75	0.52	1.00	0.39
*CYP2E1* polymorphism	0.09	0.91	0.53	0.59	0.26	0.77
Smoker habit	0.339	0.56	0.44	0.51	1.33	0.25
Occupational exposures	0.182	0.91	0.65	0.59	1.57	0.20
GST * CYP2E1	0.66	0.68	1.53	0.17	2.18	0.05
*GST* * Smoker	0.44	0.73	0.65	0.58	2.02	0.12
*GST* * different exposures	0.67	0.69	0.44	0.87	0.78	0.61
*CYP2E1* * Smoker	0.17	0.85	0.83	0.44	0.56	0.57
*CYP2E1* * different exposures	0.19	0.98	0.31	0.93	0.71	0.65
Different exposures * Smoker	1.05	0.37	1.11	0.35	0.55	0.17
Smoker * GST * CYP2E1	0.69	0.56	1.33	0.27	0.62	0.60
Different exposures * GST * CYP2E1	0.84	0.57	0.92	0.51	2.23	0.03
Different exposures * GST * Smoker	0.24	0.87	0.23	0.88	1.97	0.12
Different exposures * CYP2E1 * Smoker	1.56	0.20	0.32	0.81	1.68	0.18

N.B. A multivariate general linear model (GLM) was used; the dependent variables were normally distributed

Table 5 shows that, on using *the Kruskal–Wallis test*, there were no significant differences in the anti-CCP, CRP, and RA biomarkers between the different polymorphisms in the workers in the different sections. But in the dyeing section, the mean as well as the median of RF in the worker with *C2/C2* was much higher than those with the other two alleles, without a significant difference.

**Table 5:** Comparisons of the RA biomarkers between the workers with the different *CYP2E1* polymorphism according to their occupational task

	Anti CCP (AU/mL) Mean ± SD (Median)	CRP (mg/L) Mean ± SD (Median)	RF (IU/ml) Mean ± SD (Median)
Workers from printing Sect. (n=35)			
*C1/C1* (*n*= 17) *C1/C2*(*n*= 15) *C2/C2* (*n*=3)	10.12 ± 0.75 (9.4) 8.09 ± 0.87 (7.9) 8.57 ± 1.36 (8.9)	5.65 ± 0.52 (5.0) 4.87 ± 0.26 (5.0) 5.33 ± 1.33 (4.0)	6.12 ± 0.48 (6.0) 6.80 ± 0.33 (6.0) 6.67 ± 0.67 (6.0)
*P* value*	> 0/05	> 0/05	> 0/05
Workers from dyeing Sect. (n=87)			
*C1/C1* (*n*= 38) *C1/C2* (*n*= 39) *C2/C2* (*n*= 10)	9.73 ± 0.46 (9.3) 11.03 ± 0.70 (9.7) 10.51 ± 0.79 (10.1)	5.89 ± 0.53 (5.0) 5.72 ± 0.31 (5.0) 5.70 ± 0.50 (5.0)	6.21 ± 0.14 (6.0) 6.26 ± 0.15 (6.0) 6.90 ± 0.31 (6.5)
*P* value*	> 0/05	> 0/05	> 0/05
Workers from preparatory Sect. (n=18)			
*C1/C1* (*n*= 6) *C1/C2* (*n*= 8) *C2/C2* (*n*= 4)	12.15 ± 10.25 (11.1) 11.46 ± 1.52 (11.1) 9.21 ± 1.09 (9.3)	5.00 ± 0.26 (5.0) 5.63 ± 0.92 (5.0) 5.00 ± 0.00 (5.0)	6.33 ± 0.42 (6.0) 6.13 ± 0.23 (6.0) 6.25 ± 0.48 (6.5)
*P* value*	> 0/05	> 0/05	> 0/05

>10 is positive for Anti-CCP, CRP and RF

* Kruskal–Wallis

Table 6shows that, on using the Kruskal–Wallis test, there were no significant differences in the anti-CCP, CRP, and RA biomarkers between the different polymorphisms in the workers in the different sections. But in the dyeing section, the mean and median of CRP and RF in the worker with GSTM1 were much higher than those with the other polymorphisms, without a significant difference. 

**Table 6:** Comparisons of the RA biomarkers between the workers with the different GST polymorphism according to their occupational task

Occupation	Anti CCP (AU/mL) Mean ± SD (mean range)	CRP (mg/L) Mean ± SD (mean range)	RF (IU/ml) Mean ± SD (mean range)
Workers from printing Sect. (n=35)			
* GSTM1* (*n*= 7) *GSTT1* (*n*= 7) *GSTM1&T1* (*n*= 18) *GST NULL* (*n*= 3)	11.36 ± 1.48 (10.8) 8.69 ± 0.76 (8.1) 8.41 ± 0.79 (8.5) 9.14 ± 0.59 (9.7)	5.43 ± 0.48 (5.0) 5.29 ± 0.84 (4.0) 5.17 ± 0.42 (5.0) 5.67 ± 1.20 (5.0)	7.14 ± 0.88 (6.0) 7.43 ± 0.84 (6.0) 6.50 ± 0.19 (6.0) 8.00 ± 1.00 (7.0)
*P* value*	> 0.05	> 0.05	> 0.05
Workers from dyeing Sect. (n=87)			
* GSTM1* (*n*= 11) * GSTT1* (*n*= 8) * GSTM1&T1* (*n*= 56) * GST NULL* (*n*= 12 )	9.62 ± 0.90 (9.1) 11.14 ± 1.02 (11.1) 10.18 ± 0.40 (9.5) 11.69 ± 1.82 (9.5)	7.91 ± 1.77 (5.0) 5.13 ± 0.13 (5/0) 5.52 ± 0.20 (5.0) 5.58 ± 0.58 (5.0)	6.70± 0.31 (6.5) 6.38 ± 0.32 (6.0) 6.30 ± 0.13 (6/0) 6.00 ± 0.17 (6.0)
*P* value*	> 0.05	> 0.05	> 0.05
Workers from preparatory Sect. (n=18)			
* GSTM1* (*n*= 5) * GSTT1* (*n*= 0) * GSTM1&T1* (*n*= 9) * GST NULL* (*n*= 4)	10.07 ± 0.43 (9.8) --- 11.41 ± 1.19 (11.7) 12.11 ± 2.61 (12.4)	5.0 ± 0.00 (5.0) --- 5.67 ± 0.82 (5.0) 4.75 ± 0.25 (5.0)	6.00 ± 0.32 (6.0) --- 6.33 ± 0.33 (6.0) 6.25 ± 0.25 (6.0)
*P* value*	> 0.05	> 0.05	> 0.05

>10 is positive for Anti-CCP, CRP and RF

* Kruskal–Wallis

## Discussion

Concerning occupational exposure in the textile environment, previous studies mentioned that long-term exposure to air pollution was associated with higher risk of developing autoimmune diseases; particularly rheumatoid arthritis (RA), connective tissue diseases (CTDs) and inflammatory bowel diseases (IBD) [7, 20]. Adami et al. [20] detected the association of exposure to high concentrations of PM2.5 and PM10 and the risk of autoimmune diseases among the general population, including RA. Lei et al. [21] detected high VOCs concentrations in the urine of RA patients, which confirms the significant association of the development of RA and exposure to high concentrations of VOCs.

In the present study, the concentrations of the gases and particulate matter in different sizes was below the Egyptian limits and the international total threshold values. However, there were significant increase in the concentrations of PM10 in the preparatory section compared to the printing and dyeing sections, and in the dyeing section compared to the printing section. Also, the air levels of SO_2_ and H_2_S were significantly higher in the preparatory and dyeing sections compared to the printing section, and in the dyeing compared to the printing section whileNO_2_ was significantly higher in the printing section compared to the preparatory section. A retrospective cohort study found that RA was significantly associated with the elevation of NO_2_ exposure in Taiwan [22].

Moreover, solvents could be emitted during dyeing operations depending on the types of dyes and the processes used [23]. Also, volatile compounds are found to be emitted from drying ovens and from mineral oils used under high-temperatures for drying and preparations [10, 24]. In the present study, VOC concentrations were found to be significantly higher in the dyeing section compared to both the preparatory and printing sections, and in the preparatory section compared to the printing.

RA is a condition that affects large joints, such as the knees, elbows, hands, and wrists, and small joints, in the form of fingers and toes, and is characterized by inflammation and connective tissues damage [1, 2]. The present study revealed that complaining of joint pain was significantly higher among the exposed workers from the dyeing factory (41.4%) compared to controls (11.5%). The complaints were mainly of large joint pain in most of the exposed workers (37.9%), and only 3.6% of the workers complained of small joint pain (fingers and toes). Knee pain represented the most common joint pain among workers (24.3%). On clinical examination, knee crepitus was highly found among the complaining workers, followed by tenderness of the big joints on pressure (6.4%), and only 2 workers (1.4%) were found to have elbow crepitus. In addition, the levels of the autoimmune biomarker anti-CCP were found to be significantly higher in the exposed workers compared to their controls. Positives of CRP, RF, and ANA were also significantly higher in the exposed workers compared to the controls.

Similar results were previously detected by O’Driscoll and Mezrich [25]; they detected an increased risk of development of RA in participants exposed to high concentrations of PM2.5. In South Korea, the RA incidence rate was found to be positively correlated with PM concentrations, especially PM2.5 [26]. Too et al. [8] found an association between positive anti-CCP and exposure to textile dust. Therefore, exposure to high concentrations of PM in the present study could be considered a significant risk factor for the development of RA among the exposed workers.

The present study also revealed significantly higher levels of anti-CCP and RF in workers from the preparatory and dyeing sections compared to workers from the printing section, that could be explained by the higher concentrations of PM10 as well as VOCs in the preparatory and dyeing sections` working environment compared to that in the environment of the printing section.

A previous study suggested that autoimmune diseases are influenced by genetic, hormonal, and environmental factors [3, 4, 27]. Many VOCs are metabolized using P450 enzymes [14]. The *CYP2E1* gene is a member of the cytochrome P450 superfamily [28]. In addition, both *CYP2E1* and *GST* genes were involved in catalyzing the conjugation reaction of glutathione with electrophilic compounds. Inheritable gene alterations in *CYP2E1* or *GST* genes could cause alterations in the function of these enzymes in different human subpopulations, which may lead to differences in the metabolism of chemicals, such as VOCs [14]. Different polymorphisms have been considered as important modifiers of the individual risk for environmentally induced chronic health problems [28].

Polymorphisms of the *CYP2E1* and *GST* genes were studied in the present study for the included exposed workers and their controls. In humans, considerable inter-individual variation in the levels of P450 enzymes and their corresponding activities was reported [12]. The different genotypes may be related to the different enzymatic expression levels in individuals [29]. The present study found no significant difference in the frequency of *CYP2E1* polymorphism between the exposed workers and their controls, as well as among the exposed workers from the different occupational sections.

The *GST* genes are known as a superfamily of phase II metabolic enzymes that catalyze the detoxification of xenobiotics via glutathione conjugation. Glutathione S-transferase (GSTase) has peroxidase activity concerning cytotoxic metabolites that are produced in inflammatory reactions, which represents the main feature of RA. In addition, it has been proposed that defense mechanisms against ROS, which are impaired in RA [30]. In the present study, a significant difference was found in the distribution of *GST* polymorphisms between the exposed workers and their controls. The *GSTM1T1* polymorphism was found to be the most frequent in the exposed workers (59%), while the *GST null M1T1* was the highest among the control group (33%).

Multivariate analysis of the different risk factors in the present study revealed a significant interaction of *GST* and *CYP2E1* gene polymorphisms on RF positivity. This significant interaction increased when the effect of the difference exposures; inform of non-exposed (control subjects) and the workers from the three working sections, was added to the interaction of these two gene polymorphisms. This confirms the importance of the interaction of environmental exposures and the genes` polymorphisms, as mentioned by Arleevskaya et al. [3]. They believed that the important risk factor for RA development is a combination of genetic and environmental factors [3]. There was no significant relationship between disease risk and duration of exposure in the present study, indicating that genetic susceptibility could play a key role in the development of RA in individuals exposed to workplace pollutants, independent of exposure duration.

In our study, although RF levels showed a statistically significant difference between the control group and the exposed workers, statistical significance does not necessarily imply clinical relevance. However, a considerable proportion of the exposed workers reported joint pain. Therefore, the clinical significance of elevated RF levels may be meaningful and could have implications for workers’ health outcomes and management strategies. Therefore, there is an important need to know which polymorphism of the two genes and which occupational exposures could represent the risk for the development of RA among the textile dyeing workers. In the present study, mutant genotype *C2/C2* of the *CYP2E1* gene was found to be associated with the elevation of RF in the workers from the dyeing section in comparison to other *CYP2E1* polymorphisms. This may be attributed to the fact that the mutant *C2* allele is related to reduced *CYP2E1* enzymatic activity, as demonstrated by Ye et al. [31], and *CYP2E1* enzyme is involved in the activation of different chemicals [13]. Therefore, in the present study, the workers from the dyeing section were found to be exposed to high concentrations of PM10 and VOCs. Montero-Montoyo et al. [32] illustrated that oxidative stress due to industrial pollutants was inducing activation of P450 enzymes, particularly *CYP2E1*, increasing intracellular ROS and oxidative metabolites that lead to a loss of cell function and mutation. Moreover, Poblete-Naredo and Albores [33] demonstrated that *CYP2E1* polymorphisms can influence biomarker levels and risk profiles. Genetic variants (e.g., *C1/C2*,* C2/C2*) influence metabolic activity and hence ROS generation and oxidative stress susceptibility. Another study suggested that occupational exposures in textile settings (e.g., textile dust and dyes) had shown an increase in immune stimulation and altered immune homeostasis due to chronic irritant and oxidative stimuli [34]. In the dyeing section of the present study, workers carrying the *C2/C2* genotype of the *CYP2E1* gene exhibited higher RF levels than those with other *CYP2E1* alleles; however, the difference was not statistically significant, likely due to the small subgroup sample sizes. This suggests that workers with the *CYP2E1 C2/C2* genotype may have a greater susceptibility to RA than workers with other *CYP2E1* genotypes under the same occupational exposure conditions.

Achour et al. [4] suggested that the *GSTM1* allele encodes an active form of the glutathione S-transferase enzyme. Furthermore, differences in the frequencies of the *GST* polymorphisms exist among various ethnic populations and could account for some of the differences in disease prevalence. In the present study, CRP and RF levels were significantly higher in the workers from the dyeing section with the *GST Null* polymorphism compared to the other genotypes. The results of the present study were in contrast with the study of Achour et al. [4], who found that the deletion or Null polymorphism of *GSTT1M1* is associated with increased susceptibility to RA, where individuals who are homozygous for deletion polymorphisms in *GSTM1* and *GSTT1* do not express a functional protective protein. Previous studies on rheumatoid arthritis (RA) patients had recognized that the *GSTM1 null* genotype modulated the relationship between environmental stressors and RF production. This suggested that impaired detoxification (via *GSTM1* absence) combined with environmental oxidative stress could stimulate immune response activation and autoantibody induction [35].

The present study illustrated that concentrations of VOCs were significantly higher in the dyeing section compared to the other two sections. While the concentrations of PM_10_ were also significantly higher in the preparatory section compared to the other two sections, followed by the concentration in the dyeing section, which was significantly higher than in the printing section. The frequency of workers with the *GSTM1* genotype was found to be higher in the dyeing section than among workers from the printing and the preparatory Sect.  (77%, 29%, and 16%, respectively). Therefore, this finding may help explain the higher CRP and RF levels observed in dyeing-section workers with the *GSTM1* genotype compared with workers carrying other *GST* genotypes, although the differences were not statistically significant. The lack of significance is likely attributable to the small subgroup sample sizes in the present study, which may have reduced the statistical power to detect the expected association.

It can be concluded that the elevated PM₁₀ and VOC concentrations in the dyeing-section environment, in combination with *CYP2E1* (*C2/C2*) as well as *GSTM1* genetic polymorphisms, may be associated with higher RF levels among dyeing-section workers compared with those in the other two sections, suggesting a gene–environment interaction effect.

### Study limitations and strengths

The main limitation was to find workers who were not occupationally exposed to PAHs and VOCs and matched with the textile dyeing workers, therefore, workers from a wastewater treatment plant not occupationally exposed to these chemicals were selected to be the unexposed workers. Another limitation is that the workers were not aware of their infection history, and most of them answered that they didn’t remember.

The principal strength of this study lies in the originality of its concept. Whereas the majority of published literature has examined the association between exposure to elevated levels of air pollutants and the prevalence of rheumatoid arthritis (RA), this study uniquely explored environmental–gene interactions in the context of early prediction of RA.

## Conclusions

Environmental monitory of the air of the workplace (printing, dyeing and preparatory sections) found that the PMs with the different sizes were below the Egyptian and international standards. The concentration of PM_10_ was significantly higher in the preparatory and dyeing sections compared to the printing section, and that VOCs concentrations were significantly higher in dyeing section, followed by the preparatory section, compared to the printing section. Therefore, occupational exposure to high concentrations of PM_10_ and VOCs, mainly in the dyeing section, with the gene-gene interaction of *CYP2E1* (*C2/C2*) and *GST* (*M1* allele) polymorphisms was found to have an important association role in the elevation of RF among the workers from the dyeing section. However, further cohort studies with a larger number of workers are needed to confirm the gene-environment association with the development of RA among textile dyeing workers.

Therefore, we recommend minimizing exposure to PM and VOCs through environmental control of the workplace, including standard hygienic measures and proper ventilation, to minimize exposure to these pollutants. Furthermore, the obligation of textile dyeing workers to wear suitable protective clothing and personal protective equipment was recommended to reduce occupational health risks. In addition, pre-employment medical examination and regular follow-up must be implemented to decrease the incidence of developing RA among the workers while also providing cost-benefit advantages for the factory. Early screening of gene susceptibility through genetic counselling of *CYP2E1* and *GST* would represent a cost-effective approach for identifying workers at higher risk of occupational health hazards. Such measures would ultimately decrease the economic burden associated with losses of expert workers and the expenses of therapeutic interventions.

## Supplementary Information

Supplementary Material 1.

## Data Availability

The original data is available when requested.

## Abbreviations

ACR: American College of Rheumatology
ANA: Antinuclear antibodies
ANOVA: Analysis of variance
Anti-CCP: Anticycliccitrullinated peptides antibodies
CO: Carbon monoxide
CRP: C- Reactive Protein
CTDs: Connective tissue diseases
CYP2E1: Cytochrome P450 2E1
EDTA: Ethylene diamine tetra-acetic acid
EULAR: European Alliance of Associations for Rheumatology
GST: Glutathione S-transferase
𝐻_2_𝑆: Hydrogen sulfide
IBD: Inflammatory bowel diseases
LSD: Least Significant Differences
NH_3_: Ammonia gas
NO_2_: Nitrogen dioxide
PCR-RFLP: Polymerase chain reaction–restriction fragment length polymorphism
PM10: Inhalable Coarse Particles
PM2.5: Fine Particles
RF: Rheumatoid Factor
SD: Standard deviation
SI: Smoking index
SO2: Sulfur dioxide
TSP: Total Suspended Particulate
VOCs: Volatile organic compounds
χ^2^: Chi-square
