# Supplementary material for: Integrated evaluation of environmental monitoring, rheumatoid arthritis biomarkers, and genetic variants in textile dyeing workers
Source: J Egypt Public Health Assoc. 2026 Jun 29;101:23. doi: 10.1186/s42506-026-00217-2 (PMC13315032; doi:10.1186/s42506-026-00217-2)
Supplement: Supplementary file 1 — Supplementary Material 1. [file 42506_2026_217_MOESM1_ESM.docx]

**Supplements**

**Table (*supplement 1*): Comparisons between the air pollutants (particulates and gases) in the different occupational areas in the factory**

|  | **Printing section** | | **Dyeing section** | | **Preparatory section** | | ANOVA | | EEAA, 2015 | ACGIH TLV 8-hour TWA | OSHA- PEL 8-hour TWA |
| --- | --- | --- | --- | --- | --- | --- | --- | --- | --- | --- | --- |
|  | Mean | SE | Mean | SE | Mean | SE | F-ratio | P-value |  |  |  |
| **Particulate Matters (PM)** | | | | | | | | | | | |
| PM2.5 | 0.20 | 0.03 | 0.13 | 0,01 | 0.88 | 0.40 | 2.274 | 0.145 | 3.0 | 10.0 | 15.0 |
| PM10 | 0.33^(a,b)^ | 0.04 | 0.41^(c)^ | 0.06 | 1.41 | 0.43 | 4.166 | 0.042 | 3.0 |  |  |
| TSP | 0.45 | 0.05 | 0.53 | 0.09 | 1.44 | 0.59 | 1.857 | 0.198 | 10.0 |  |  |
| **Gases** | | | | | | | | | | | |
| VOCs | 42.40 ^(a,b)^ | 14.56 | 113.5^(c)^ | 9.35 | 79.5 | 2.39 | 12.461 | 0.001 |  |  |  |
| NO2 | 0.28 | 0.10 | 0.26 | 0.02 | 0.16 | 0.01 | 2.954 | 0.091 | 5.6 | 0.38 | 1.88 |
| SO2 | 0.13 ^(a,b)^ | 0.02 | 0.28^(c)^ | 0.03 | 0.39^(a)^ | 0.06 | 8.216 | 0.006 | 5.2 | 0.66 |  |
| NH3 | 0.19 | 0.02 | 0.25 | 0.03 | 0.21 | 0.01 | 1.908 | 0.191 |  |  |  |
| CO | 1.23 | 0.71 | 0.33 | 0.06 | 0.69 | 0.22 | .969 | 0.407 | 290.0 | 28.64 | 57.28 |
| H2S | 0.19 ^(a,b)^ | 0.05 | 0.08 | 0.02 | 0.06 | 0.01 | 5.208 | 0.024 |  |  |  |

Egyptian guideline in workshop environment (exposure for 8 hours) (EEAA, 2015), ACGIH TLV 8-hour TWA (Frampton et al., 2002), OSHA- PEL 8-hour TWA (OSHA, 1991), American Conference of Governmental Industrial Hygienists (ACGIH), Occupational Safety and Health Administration (OSHA), Threshold Limit Values (TLVs), Time-weighted average (TWA), Permissible Exposure Limit (PEL)

*N.B.: using LSD (a): significant different between printing and preparatory, (b): significant different between printing and dyeing, (c): significant different between dyeing and preparatory*

**Figures**
